# *FLT4* Mutations Are Associated with Segmental Lymphatic Dysfunction and Initial Lymphatic Aplasia in Patients with Milroy Disease

**DOI:** 10.3390/genes12101611

**Published:** 2021-10-13

**Authors:** Ningfei Liu, Minzhe Gao

**Affiliations:** Department of Plastic & Reconstructive Surgery, Shanghai Ninth People’s Hospital, Shanghai Jiao Tong University School of Medicine, Shanghai 200011, China; sherryg95@163.com

**Keywords:** *FLT4*, VEGFR3, Milroy disease (MD), lymphedema, lymphatic dysfunction, initial lymphatic aplasia

## Abstract

This study explored mutations in the Fms-related tyrosine kinase 4/vascular endothelial growth factor receptor 3 gene (*FLT4*) and lymphatic defects in patients with Milroy disease (MD). Twenty-nine patients with lower limb lymphedema were enrolled. Sixteen patients had a familial history of MD, while 13 patients exhibited sporadic MD. Clinical signs, *FLT4* mutations, indocyanine green (ICG) lymphography findings, and skin tissue immunohistochemical staining results were evaluated. Twenty-eight variants in *FLT4* were identified. Twelve of these have previously been reported, while 16 are novel. Of the 28 variants, 26 are missense mutations, and the remaining two comprise a splicing mutation and a non-frame shift mutation. Twenty-five variants are located in the intracellular protein tyrosine kinase domain; three are located in the extracellular immunoglobulin domain. Substantially delayed contrast-enhanced tortuous lymphatic vessels were visualized to the ankle or knee level in 15 of 23 patients who underwent ICG lymphography. No initial lymphatic vessels were visualized in skin specimens from four patients who did not exhibit lymphatic vessels during imaging analyses. No specific variant was identified in relation to the unique clinical phenotype. Segmental dysfunction of lymphatic vessels and initial lymphatic aplasia are present in MD patients with *FLT4* mutations.

## 1. Introduction

Milroy disease (MD; MIM 153100) is a rare, congenital onset, autosomal, dominantly inherited condition associated with primary lymphedema. The gene encoding Fms-related tyrosine kinase (*FLT4*), also known as vascular endothelial growth factor receptor 3 (VEGFR3) is the only known causative gene for MD [1], and *FLT4* mutations are responsible for the majority of MD cases. MD most commonly occurs bilaterally in the lower limbs and is rarely seen in a single leg. In most patients, swelling is pronounced in the dorsum of the foot with fibrotic thickening of the toe skin. Swelling may also extend to the ankle or knee level. Other clinical signs include a prominent great saphenous vein, hydrocoeles, and deep venous insufficiency. Clinical manifestations of MD exhibit considerable variability [2], including within families where affected individuals carry the same mutation. However, de novo cases have been observed in isolated patients; therefore, a family history is not essential for diagnosis [3,4,5]. MD is regarded as a disease caused by lymphatic dysfunction because lymphatic vessels were not visible in most tested patients during lymphoscintigraphic examination; this phenomenon is known as “functional aplasia” because it involves failed isotopic tracer uptake by lymphatic capillaries [6,7]. However, different results were observed in some patients who exhibited functional lymphatic vessels in the affected legs during the same imaging test [7]. These variable clinical manifestations suggest a complex pathogenesis and complicated underlying pathophysiology.

Published reports have described various mutations in *FLT4* in probands and families with MD. Most reported mutations are missense variants; some comprise in-frame indels located in the VEGFR3 protein kinase domain alone [7].

This study presents *FLT4* mutation findings from whole-exome sequencing (WES) analysis of a cohort of 29 MD patients. The patients underwent indocyanine green (ICG) lymphography examination to localize and characterize lymphatic defects in their affected limbs. For patients who failed to demonstrate lymphatic vessels during ICG lymphography examination, skin biopsies were performed to identify initial lymphatic vessels in the skin. By combined analysis of *FLT4* mutation assessment, functional and structural lymphatic imaging examinations, and skin lymphatic histology assessments, we sought to establish genotype–phenotype relationships in patients with MD.

## 2. Materials and Methods

### 2.1. Patient Recruitment and Ethical Approval

In total, 29 patients with congenital MD, who visited our clinic between 1 January 2010 and 30 December 2020, were enrolled in the study. Sixteen patients had a familial history of MD, while 13 patients had sporadic MD. MD was diagnosed on the basis of a patient’s clinical history and physical examination findings, as well as WES of blood samples with identified *FLT4* variants. ICG lymphography was performed in 23 patients. Full-thickness skin biopsies and immunohistochemical staining for lymphatic evaluation of the lymphedematous limbs were performed in five patients. This clinical trial was approved by the Ethics Committee of the Shanghai Ninth People’s Hospital (Ethical Approval Code: 201428); all patients provided written informed consent for inclusion in the study.

### 2.2. Whole-Exome Sequencing

DNA was isolated from blood that had been collected from 16 probands with family histories, as well as their family members and 13 isolated probands. WES was performed using the Agilent SureSelectXT Human All Exon V6 kit (Agilent Technologies, Santa Clara, CA, USA) and sequenced with an Illumina HiSeq sequencing system (Illumina Inc., San Diego, CA, USA). Sequences were aligned to the human reference genome *(Hg19)* using the Burrows–Wheeler Aligner (version 0.7.17); variants were called with SAMtools and the Genome Analysis Toolkit (version 4.0) and annotated with ANNOVAR software [8]. Variants with a quality score of <20 were excluded from analysis. Perl scripts were used to filter single-nucleotide variants with respect to variants present in the dbSNP150 database. A benign polymorphism was defined as any single nucleotide variant recorded in dbSNP150 that exhibited a minor allele frequency of ≥1% in the Exome Aggregation Consortium or Genome Aggregation Database; all benign polymorphisms were removed before subsequent analysis. PolyPhen2, SIFT, MutationTaster, CADD and Dann were used to predict mutation functional importance. *FLT4* variants were annotated with InterVar command line driven software (Version 2.1.2) using default options, which is based on ANNOVAR software. This estimates the clinical impact of variants based on guidelines for variant interpretations from the American College of Medical Genetics and Genomics and the Association for Molecular Pathology in clinical sequencing.

### 2.3. ICG Lymphography

ICG lymphography examinations were performed in 23 patients. After intradermally injecting the contrast agent ICG (2.5 mg/mL) into the toe web spaces (two to three points per limb, 0.05 mL per point), a photodynamic eye fluorescence-locating instrument (Hamamatsu Photonics, Hamamatsu, Japan) was used for the dynamic observation of lymph flow immediately and 120 min after injection.

Injections were added above the ankle and knee in two patients in whom the lymphatic flow was not visualized in the affected dorsum of the foot to explore the extent of functionally affected lymphatic vessel territory.

### 2.4. Immunohistochemical Staining

Immunohistochemical staining was performed on whole-thickness skin sections (2 × 1 cm^2^) from the lymphedematous feet of five patients. Lymphatic and vascular staining was performed using primary anti-podoplanin (1:50; AngioBio, San Diego, CA, USA) and rabbit anti-human CD-31 antibodies (1:200 Abcam, Cambridge, UK); Secondary antibodies Alexa Fluor555 goat anti-mouse (1:300 Invitrogen, San Diego, CA, USA) and Alexa Fluor 488 goat anti-rabbit antibodies (1:300 Invitrogen) were used to visualize the signal. Photography and whole-slide image construction were performed using a confocal microscope (LSM 710; Carl Zeiss, Jena, Germany).

## 3. Results

The study population comprised 14 male patients and 15 female patients (mean age, 14.1 years; range, 1 month to 28 years). Twenty-eight patients had bilateral leg lymphedema and one patient had left leg lymphedema. Ten patients had edema limited to the dorsa of both feet. Eleven patients had extended swelling below the knee. In the remaining patients, swelling was obvious in one foot; it extended beyond the ankle or up to knee in the other leg. Hydrocele was evident in two patients; a cnemis constriction band was found in two patients; and a prominent great saphenous vein was visible in four patients. No pitting edema was evident in any patient, including the infant. Three patients had deep venous reflux. Upslanting toenails and papillomatosis were seen in five patients. Patients’ clinical signs, imaging findings, and genetic findings are summarized in Table 1.

### 3.1. Whole-Exome Sequencing

**Figure 1:**
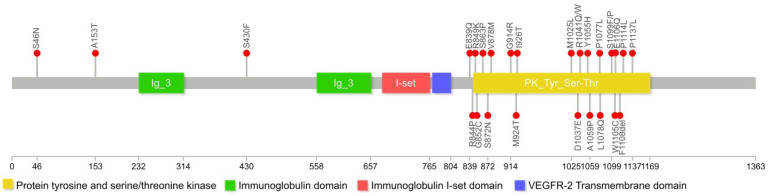
Schematic representation of *FLT4* gene and positions of mutations identified in patients with MD in this study. Variants were located in the tyrosine kinase and immunoglobulin domains.

WES identified 28 *FLT4* variants among our patients (Table 1, Figure 1). Twelve of these have previously been reported in the human gene mutation database (HGMD ^®^); all comprised missense variants located in the intracellular tyrosine kinase domain (exons 17, 18, 19, 20, 23, and 25). Sixteen variants are novel. Among the 28 variants, 26 were missense mutations, while the remaining two were a splicing mutation and a nonframeshift mutation. We observed three variants in the extracellular immunoglobulin domain (exons 2 and 10) in one patient with familial MD and two patients with sporadic disease. Notably, one patient carried two variants: one in each of the protein kinase and immunoglobulin domains; another patient carried two variants: both in the protein kinase domain. To our knowledge, this is the first report of *FLT4/VEFDR3* variants found outside the tyrosine kinase domain in patients with MD. Variant annotation results are shown in Table 1 and Table S1. Nineteen variants were likely pathogenic and nine were of uncertain significancy (VUS). Nine VUS were predicted to be pathogenic by SIFT, POLYPhen or Mutationtaster, and among them four were previously reported as pathogenicity [1,4,7,9]. Combined with our clinical findings and the results predicted by the software, these 9 VUS are more likely to be pathogenic mutations.

### 3.2. ICG Lymphography

**Figure 2:**
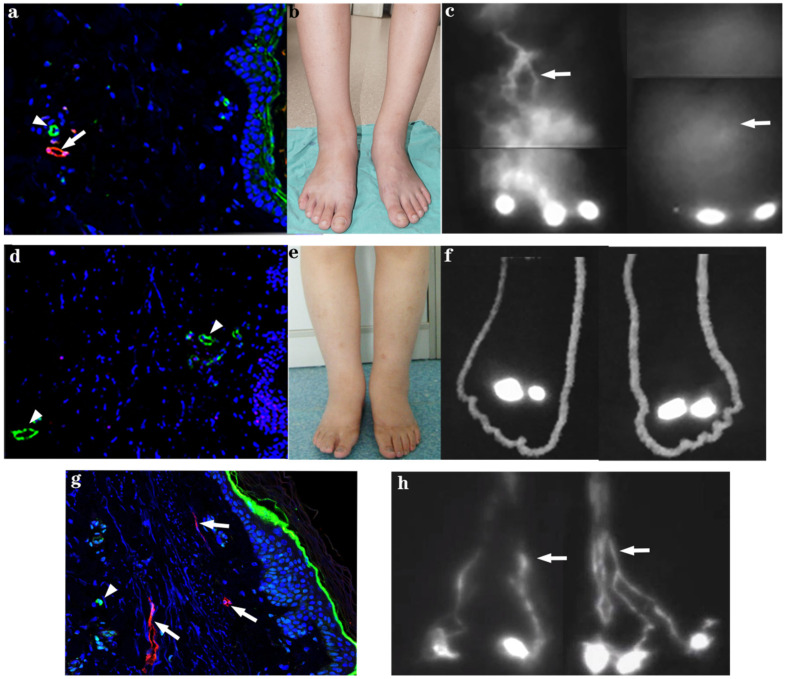
Clinical phenotype, corresponding skin histology, and ICG lymphography. Top: Female patient with isolated MD (S3 in Table 1). (**a**) Dermal initial lymphatic vessel (arrows) and blood capillaries (arrowheads) in the skin. (**b**) Bilateral lymphedema of the legs. (**c**) Tortuous and irregular lymphatic vessels (arrows) in the dorsum of the right feet and lower leg and massive contrast diffusion in the left lower leg. Middle: (**d**) Female patient with familial MD (F5 in Table 1). (**d**) No lymphatic vessels were identified and only blood capillaries were detected (arrowheads) in the dorsum skin of the patient. (**e**) Bilateral lymphedema below the knee. (**f**) No enhanced lymphatic vessels were visualized on indocyanine green lymphography. Bottom: (**g**) Dermal initial lymphatic vessel (arrows) and blood capillaries (arrowhead) in normal control skin. (**h**) lymph collectors (arrows) displayed in bilateral dorsum of foot and lower legs of normal control.

Twenty-three patients underwent ICG lymphography examination. Immediately or within a few minutes after ICG injection, enhanced lymphatic collectors could be clearly delineated from the injection site and drained upward in healthy limbs toward the lymph nodes in the groin [10,11]. Substantially delayed contrast-enhanced lymphatic vessels were visualized in 15 of 23 (65%) tested patients, compared with normal limbs. Immediately after injection, only a small subset of lymphatic vessels was visible extending from the injection point. At 2 h after contrast injection, tortuous, irregular, and network-like lymphatic vessels were visible in the dorsum/sole and below the ankle in both limbs in eight patients (Figure 2); in five patients, tortuous lymphatic vessels slowly traveled upward and stopped at the knee level in the affected legs. In three patients, lymphatic vessels were visible in one lower leg below the ankle, but were absent from the other leg. This gradual enhancement of collecting lymphatic vessels in the distal part of the leg may have been caused by altered contrast uptake or transportation by the initial lymphatic vessels and lymphatic collectors. No contrast-enhanced lymphatic vessels were visible 2 h after contrast injection in the dorsum of the foot in eight patients. This was probably caused by the failed uptake of injected fluorescent contrast by initial lymphatic vessels because of functional or structural defects in lymphedematous skin. In two of these eight patients, 2–3 lymphatic collectors were visible above the knee (Figure 2) after additional contrast injection in the ankle and knee. This suggests a localized absence of functional peripheral lymphatic vessels within the diseased limb. Notably, one proband exhibited no contrast-enhanced lymphatic vessels in either affected limb during ICG lymphography examination; his father and grandfather had tortuous and irregular lymphatic vessels in both affected legs.

### 3.3. Immunohistochemical Staining

Initial lymphatic vessels were visualized in lymphedematous skin samples from one patient in whom ICG lymphography showed slowly enhanced tortuous lymphatic vessels in the lower leg. The lumens of these vessels were wider compared with that in normal skin (Figure 2a–c,g). No podoplanin-positive lymphatic vessels were identified in the dermis or subcutaneous tissue following dorsum biopsy in four patients who had not exhibited lymphatic vessels 2 h after contrast injection (Figure 2d–f). These histology findings suggest the presence of initial lymphatic aplasia in affected patients.

## 4. Discussion

All previously reported *FLT4* mutations in patients with MD were located in protein tyrosine kinase domains 1 or 2 (TK1 and TK2); exons 17–20 and 22–26, respectively) [7]; these mutations reduce tyrosine kinase activity and affect lymphatic development [12]. Here, we used WES to identify 28 *FLT4* variants, including 16 novel ones in patients with MD., Twenty-six of these variants are located in TK1 and TK2. Considering previously reported findings, more than 60 variants in *FLT4* have been discovered thus far, which implies considerable mechanistic heterogeneity in MD. Importantly, we identified two novel variants in the extracellular immunoglobulin domain, which to our knowledge is the first report of an *FLT4* variant in this domain in patients with MD. Thus far, deleterious mutations in the *FLT4* immunoglobulin domain were only reported in patients with the most common cyanotic congenital heart disease (Tetralogy of Fallot) [13], who did not also appear to have MD. Our study shows that the *FLT4* mutation in MD is not limited to TK1 or TK2, but may also occur in the immunoglobulin domain. The underlying mechanism of this, and how mutations interfere with the protein function, should be investigated in future studies.

In previous reports, functional lymphatic vessels could not be identified in the legs of most patients with MD [2,3,7], conversely, our study revealed that most patients had lymphatic collectors with considerably slowed transport function in the distal part of affected legs. This finding was achieved because the ICG lymphatic scan method allows direct dynamic observation from the contrast injection point on the dorsum of the foot. The characteristics of partial and delayed visualization of the lymphatic vessels likely involve capillary or collecting lymphatic dysfunctions in the formation and transportation of lymph. Contrast-enhanced lymphatic vessels extended to the ankle or knee level of our MD patients, consistent with the extent of swelling in affected legs.

In four patients who did not exhibit lymphatic vessels in the affected legs during imaging analyses, the histological examination of biopsy tissue from the dorsum of the foot revealed no podoplanin-positive lymphatic vessels; lymphatic capillaries were identified in the superficial or deep dermis or in subcutaneous tissue. In contrast lymphatic vessels were clearly visible in the skin tissue of a patient who with slowly enhanced tortuous lymphatic vessels. Thus, our histological results suggest initial lymphatic vessel aplasia is present in some MD patients [14,15].

By combining WES, lymphatic imaging examinations, and skin histology assessments, we assessed genotype–phenotype relationships in our MD patients. Among patients with ICG lymphography-confirmed lymphatic dysfunction, there were nine variants located in exons 9, 17, 18, 19, 20, 23, 24, and 25 of *FLT4* (TK1 and TK2); one variant was identified in exon 10 (the immunoglobulin domain). However, three missense mutations in exons 17, 18, and 23 of *FLT4* were also identified in patients with lymphatic aplasia who exhibited no initial lymphatic vessels in the affected skin. Thus, in patients with exonic variants, there were overlaps between the two types of lymphatic defects: lymphatic dysfunction and initial lymphatic aplasia. In particular, a similar lymphatic dysfunction phenomenon (i.e., tortuous lymphatic vessels with considerably slowed transport function) was observed in patients with variants in both the immunoglobulin and protein kinase domains.

**Figure 3:**
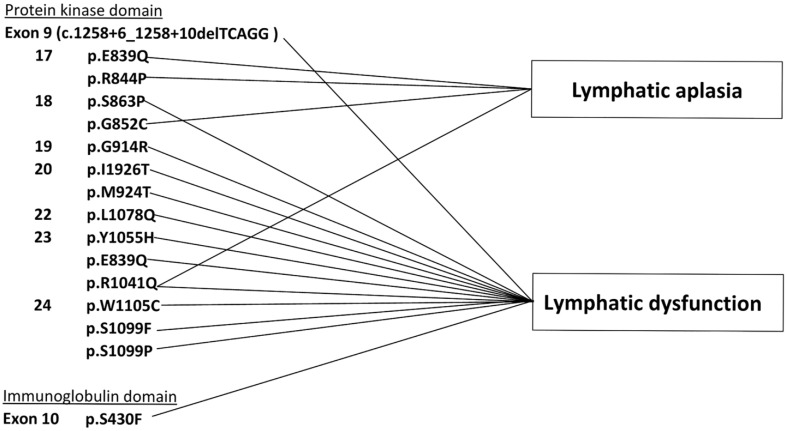
Schematic depiction of *FLT4* mutations and corresponding lymphatic malformations among patients in this study.

Furthermore, three patients all showed the same mutation in exon 23 (c.3122G>A); two of these patients had lymphatic dysfunction on their lymph scans, while the third had dermal capillary lymphatic aplasia (Figure 3). Therefore, we could not distinguish the impact of variants in these two functional domains with respect to lymphatic defects, nor could we identify specific variants related to unique clinical phenotypes based on our collected data.

The present study demonstrated heterogeneous lymphatic defects in patients with MD who exhibited *FLT4* mutations. Lymphatic malformations affect either lymphatic capillaries or lymphatic collectors; functional defects may cause initial and collecting vessel transportation failure or lymphatic capillary sprouting failure during the embryonic stage, leading to skin lymphatic capillary aplasia. The diverse pathological changes of lymphatic vessels observed in MD patients with *FLT4* mutations suggest that this gene has multiple roles in both lymphatic function and lymphangiogenesis [16,17]. It is also conceivable that other genes modify the effects of *FLT4*. Further investigations of embryonic lymphatic system development will enable a better understanding of the precise pathogenesis mechanism related to *FLT4* mutations and the relationships between genotype and phenotype in patients with MD.

## 5. Conclusions

This study is the first time to identify mutation in the immunoglobulin domain of FLT4 in patients with MD. It also shows that *FLT4* mutations are associated with two types of lymphatic defects in patients with MD. One is lymphatic collector dysfunction which appears to be a common lymphatic pathology in MD, and the other is initial lymphatic aplasia in the skin. Moreover, lymphatic dysfunction was associated with mutations in both the immunoglobulin and protein kinase domains. Because of the heterogeneous lymphatic vessel pathology in MD, its treatment may require multiple therapeutic approaches.

## Figures and Tables

**Table 1:** Genetic & Clinical Findings of 29 MD patients with *FLT4* Mutations.

Patient ID	Exon	cDNA	Protein	Pathogenicity	Interpro Domain	Gender	Extent of Swelling	ICG Lymphgraphy Scan Findings	Skin Histology	Other Symptoms
F1	23 *	c.3163G>C	p.Y1055H	LP	PKD	Female	Below ankle	Lymphatics below ankle	ND	
F2	17	c.2531G>C	p.R844P	LP	PKD	Male	Below knee	-	No lymphatic in the skin	Prominent vein
F3	18	c.2632G>A	p.V878M	LP	PKD	Female	Below ankle	ND	ND	Prominent vein
F4	25	c.3410C>T	p.P1137L	VUS	PKD	Male	Below knee	-	ND	
F5	23	c.3122G>A	p.R1041Q	LP	PKD	Female	Below ankle	-	No lymphatic in the skin	
F6	23	c.3122G>A	p.R1041Q	LP	PKD	Female	Below knee	Lymphatic below knee	ND	Deep venous reflux
F7	23	c.3122G>A	p.R1041Q	LP	PKD	Female	Below ankle	Lymphatic below ankle	ND	
F8	23	c.3121C>T	p.R1041W	LP	PKD	Female	Below knee	ND	ND	
F9	24 *	c.3315G>C	p.W1105C	VUS	PKD	Male	Below ankle	Lymphatic below knee	ND	cnemis constriction band
F10	24 *	c.3295T>C	p.S1099P	LP	PKD	Male	Below ankle	Lymphatic below ankle	ND	
F11	9 *	c.1258+6_1258+10delTCAGG		VUS		Female	Below ankle (left)	lymphatic below ankle (left)	ND	
F12	17 *	c.2515G>C	p.E839Q	LP	PKD	Female	Below knee	**-**	No lymphatic in the skin	
F13	10 *	c.1289C>T	p.S430F	VUS	IgD	Male	Below ankle (left)	Lymphatic below ankle	ND	Hydroceles, cnemis constriction band
F14	23	c.3111C>G	p.D1037E	LP	PKD	Female	Below knee	ND	ND	
F15	20 *	c.2771T>C	p.M924T	VUS	PKD	Male	Below knee	Lymphatic below knee	ND	Deep venous reflux
F16	24 *	c.3296C>T	p.S1099F	LP	PKD	Male	Below ankle	lymphatic below ankle (left)	ND	
								**-** (right)		
**S1 ^△^**	24 *	c.3230C>T	p.P1077L	LP	PKD	Male	Below knee	**-**	ND	
	18 *	c.2615G>A	p.S872N	LP	PKD					
S2	24 *	c.3233T>A	p.L1978Q	LP	PKD	Female	Below ankle	Lymphatic below ankle (left)	ND	
								**-** (right)		
S3	23 *	c.3175G>C	p.A1059P	LP	PKD	Female	Below knee	Lymphatic below knee	Lymphatic visualized in the skin	
S4	25	c.3341C>T	p.P1114L	VUS	PKD	Male	Below knee	ND	ND	Hydroceles
S5	24 *	c.3316G>C	p.E1106Q	LP	PKD	Male	Below ankle	**-**	ND	
S6	18	c.2587T>C	p.S863P	LP	PKD	Male	Below knee	Lymphatic below knee	ND	
S7	18 *	c.2546G>A	p.R849K	VUS	PKD	Male	Below ankle	ND	ND	Prominent vein
S8	18	c.2554G>T	p.G852C	LP	PKD	Female	Below ankle	-	No lymphatic in the skin	
**S9 ^△^**	19	c.2740G>C	p.G914R	LP	PKD	Female	Below ankle	Lymphatic below ankle (left)	ND	
	10 *	c.1289C>T	p.S430F	VUS	IgD			**-** (right)		
S10	24	c.3323_3325de	p.F1108del	LP		Female	Below knee	**-**	ND	Prominent vein
S11	20	c.2777T>C	p.I926T	VUS	PKD	Male	Below ankle	Lymphatic below ankle	ND	Deep venous reflux
S12	2 *	c.137G>A	p.S46N	VUS	IgD	Female	Below knee	ND	ND	
S13	22 *	c.3073A>T	p.M1025L	LP	PKD	Male	Below ankle	ND	ND	

**^△^**: Patients carrying 2 variants *: Novel variants; F: Familiar case; S: Sporatic case; PKD: Protein kinase domain; IgD: Immunoglobulin domain; VUS: Variants uncertain significance; LP: Likely pathogenic; ND: Not done.

## Data Availability

The data presented in this study are available on request from the corresponding author.

## Supplementary Materials

The following are available online at www.mdpi.com/article/10.3390/genes12101611/s1, Table S1: *FLT4* variants annotation results.
