# Supplementary material for: FLT4 Mutations Are Associated with Segmental Lymphatic Dysfunction and Initial Lymphatic Aplasia in Patients with Milroy Disease
Source: Genes (Basel). 2021 Oct 13;12(10):1611. doi: 10.3390/genes12101611 (PMC8535675; doi:10.3390/genes12101611)
Supplement: Supplementary file 1 [file genes-12-01611-s001.zip › genes-1358529-supplementary.pdf]

genes

MDPI

Article

FLT4 Mutations Are Associated with Segmental Lymphatic Dysfunction and Initial Lymphatic Aplasia in Patients with Milroy Disease

Ningfei Liu * and Minzhe Gao

Department of Plastic & Reconstructive Surgery, Shanghai Ninth People's Hospital, Shanghai Jiao Tong University School of Medicine, Shanghai, 200011, China
*Correspondence: liuningfei@126.com; Tel.: +86-21-23271699 (ext. 5734); Fax: +86-21-53078128

Supplementary Materials

Table S1. FLT4 Variants annotation result.

(NM_182925)	cDNA	Protein	Intervar	detail
2	c.137G>A	p.S46N	VUS	PVS1=0 PS=[0, 0, 0, 0, 0] PM=[0, 1, 0, 0, 0, 0, 0] PP=[0, 0, 0, 1, 0, 0] BA1=0 BS=[0, 0, 0, 0, 0] BP=[0, 0, 0, 0, 0, 0, 0, 0]
9	c.1258+6_1258+10delTCAGG	-	VUS	PVS1=0 PS=[0, 0, 0, 0, 0] PM=[0, 1, 0, 0, 0, 0, 0] PP=[0, 0, 0, 1, 0, 0] BA1=0 BS=[0, 0, 0, 0, 0] BP=[0, 0, 0, 0, 0, 0, 0, 0]
10	c.1289C>T	p.S430F	VUS	PVS1=0 PS=[0, 0, 0, 0, 0] PM=[0, 1, 0, 0, 0, 0, 0] PP=[0, 0, 1, 1, 0, 0] BA1=0 BS=[0, 0, 0, 0, 0] BP=[0, 0, 0, 0, 0, 0, 0, 0]
17	c.2515G>C	p.E839Q	Likely pathogenic	PVS1=0 PS=[0, 0, 0, 0, 0] PM=[1, 1, 0, 0, 0, 0, 0] PP=[0, 0, 1, 1, 0, 0] BA1=0 BS=[0, 0, 0, 0, 0] BP=[0, 0, 0, 0, 0, 0, 0, 0]
17	c.2531G>C	p.R844P	Likely pathogenic	PVS1=0 PS=[0, 0, 0, 0, 0] PM=[1, 1, 0, 0, 0, 0, 0] PP=[0, 0, 1, 1, 1, 0] BA1=0 BS=[0, 0, 0, 0, 0] BP=[0, 0, 0, 0, 0, 0, 0, 0]
18	c.2546G>A	p.R849K	VUS	PVS1=0 PS=[0, 0, 0, 0, 0] PM=[0, 1, 0, 0, 0, 0, 0] PP=[0, 0, 0, 1, 0, 0] BA1=0 BS=[0, 1, 0, 0, 0] BP=[0, 0, 0, 0, 0, 0, 0, 0]
18	c.2554G>T	p.G852C	Likely pathogenic	PVS1=0 PS=[0, 0, 0, 0, 0] PM=[1, 1, 0, 0, 0, 0, 0] PP=[0, 0, 1, 1, 1, 0] BA1=0 BS=[0, 0, 0, 0, 0] BP=[0, 0, 0, 0, 0, 0, 0, 0]
18	c.2587T>C	p.S863P	Likely pathogenic	PVS1=0 PS=[0, 0, 0, 0, 0] PM=[1, 1, 0, 0, 0, 0, 0] PP=[0, 0, 1, 1, 1, 0] BA1=0 BS=[0, 0, 0, 0, 0] BP=[0, 0, 0, 0, 0, 0, 0, 0]
18	c.2615G>A	p.S872N	Likely pathogenic	PVS1=0 PS=[0, 0, 0, 0, 0] PM=[1, 1, 0, 0, 0, 0, 0] PP=[0, 0, 1, 1, 0, 0] BA1=0 BS=[0, 0, 0, 0, 0] BP=[0, 0, 0, 0, 0, 0, 0, 0]
18	c.2632G>A	p.V878M	Likely pathogenic	PVS1=0 PS=[0, 0, 0, 0, 0] PM=[1, 1, 0, 0, 0, 0, 0] PP=[0, 0, 1, 1, 1, 0] BA1=0 BS=[0, 0, 0, 0, 0] BP=[0, 0, 0, 0, 0, 0, 0, 0]
19	c.2740G>C	p.G914R	Likely pathogenic	PVS1=0 PS=[0, 0, 0, 0, 0] PM=[1, 1, 0, 0, 0, 0, 0] PP=[0, 0, 1, 1, 0, 0] BA1=0 BS=[0, 0, 0, 0, 0] BP=[0, 0, 0, 0, 0, 0, 0, 0]
20	c.2771T>C	p.M924T	VUS	PVS1=0 PS=[0, 0, 0, 0, 0] PM=[0, 1, 0, 0, 0, 0, 0] PP=[0, 0, 1, 1, 0, 0] BA1=0 BS=[0, 0, 0, 0, 0] BP=[0, 0, 0, 0, 0, 0, 0, 0]
20	c.2777T>C	p.I926T	VUS	PVS1=0 PS=[0, 0, 0, 0, 0] PM=[0, 1, 0, 0, 0, 0, 0] PP=[0, 0, 1, 1, 1, 0] BA1=0 BS=[0, 0, 0, 0, 0] BP=[0, 0, 0, 0, 0, 0, 0, 0]

22	c.3073A>T	p.M1025L	Likely pathogenic	PVS1=0 PS=[0, 0, 0, 0, 0] PM=[1, 1, 0, 0, 0, 0, 0] PP=[0, 0, 1, 1, 0, 0] BA1=0 BS=[0, 0, 0, 0, 0] BP=[0, 0, 0, 0, 0, 0, 0, 0]
23	c.3111C>G	p.D1037E	Likely pathogenic	PVS1=0 PS=[0, 0, 0, 0, 0] PM=[1, 1, 0, 0, 0, 0, 0] PP=[0, 0, 1, 1, 0, 0] BA1=0 BS=[0, 0, 0, 0, 0] BP=[0, 0, 0, 0, 0, 0, 0, 0]
23	c.3121C>T	p.R1041W	Likely pathogenic	PVS1=0 PS=[0, 0, 0, 0, 0] PM=[1, 1, 0, 0, 0, 0, 0] PP=[0, 0, 1, 1, 1, 0] BA1=0 BS=[0, 0, 0, 0, 0] BP=[0, 0, 0, 0, 0, 0, 0, 0]
23	c.3122G>A	p.R1041Q	Likely pathogenic	PVS1=0 PS=[0, 0, 0, 0, 0] PM=[1, 1, 0, 0, 1, 0, 0] PP=[0, 0, 1, 1, 1, 0] BA1=0 BS=[0, 0, 0, 0, 0] BP=[0, 0, 0, 0, 0, 0, 0, 0]
23	c.3163G>C	p.Y1055H	Likely pathogenic	PVS1=0 PS=[0, 0, 0, 0, 0] PM=[1, 1, 0, 0, 0, 0, 0] PP=[0, 0, 1, 1, 0, 0] BA1=0 BS=[0, 0, 0, 0, 0] BP=[0, 0, 0, 0, 0, 0, 0, 0]
23	c.3175G>C	p.A1059P	Likely pathogenic	PVS1=0 PS=[0, 0, 0, 0, 0] PM=[1, 1, 0, 0, 0, 0, 0] PP=[0, 0, 1, 1, 0, 0] BA1=0 BS=[0, 0, 0, 0, 0] BP=[0, 0, 0, 0, 0, 0, 0, 0]
24	c.3230C>T	p.P1077L	Likely pathogenic	PVS1=0 PS=[0, 0, 0, 0, 0] PM=[1, 1, 0, 0, 0, 0, 0] PP=[0, 0, 1, 1, 0, 0] BA1=0 BS=[0, 0, 0, 0, 0] BP=[0, 0, 0, 0, 0, 0, 0, 0]
24	c.3233T>A	p.L1078Q	Likely pathogenic	PVS1=0 PS=[0, 0, 0, 0, 0] PM=[1, 1, 0, 0, 0, 0, 0] PP=[0, 0, 1, 1, 0, 0] BA1=0 BS=[0, 0, 0, 0, 0] BP=[0, 0, 0, 0, 0, 0, 0, 0]
24	c.3295T>C	p.S1099P	Likely pathogenic	PVS1=0 PS=[0, 0, 0, 0, 0] PM=[1, 1, 0, 0, 0, 0, 0] PP=[0, 0, 1, 1, 0, 0] BA1=0 BS=[0, 0, 0, 0, 0] BP=[0, 0, 0, 0, 0, 0, 0, 0]
24	c.3296C>T	p.S1099F	Likely pathogenic	PVS1=0 PS=[0, 0, 0, 0, 0] PM=[1, 1, 0, 0, 0, 0, 0] PP=[0, 0, 1, 1, 0, 0] BA1=0 BS=[0, 0, 0, 0, 0] BP=[0, 0, 0, 0, 0, 0, 0, 0]
24	c.3315G>C	p.W1105C	VUS	PVS1=0 PS=[0, 0, 0, 0, 0] PM=[0, 1, 0, 0, 0, 0, 0] PP=[0, 0, 1, 1, 0, 0] BA1=0 BS=[0, 0, 0, 0, 0] BP=[0, 0, 0, 0, 0, 0, 0, 0]
24	c.3316G>C	p.E1106Q	Likely pathogenic	PVS1=0 PS=[0, 0, 0, 0, 0] PM=[0, 1, 0, 0, 1, 0, 0] PP=[0, 0, 1, 1, 0, 0] BA1=0 BS=[0, 0, 0, 0, 0] BP=[0, 0, 0, 0, 0, 0, 0, 0]
24	c.3323_3325del	p.1108_1109del	Likely pathogenic	PVS1=0 PS=[0, 0, 0, 0, 0] PM=[0, 1, 0, 1, 0, 0, 0] PP=[0, 0, 0, 1, 1, 0] BA1=0 BS=[0, 0, 0, 0, 0] BP=[0, 0, 0, 0, 0, 0, 0, 0]
25	c.3341C>T	p.P1114L	VUS	PVS1=0 PS=[0, 0, 0, 0, 0] PM=[0, 1, 0, 0, 0, 0, 0] PP=[0, 0, 1, 1, 1, 0] BA1=0 BS=[0, 0, 0, 0, 0] BP=[0, 0, 0, 0, 0, 0, 0, 0]
25	c.3410C>T	p.P1137L	VUS	PVS1=0 PS=[0, 0, 0, 0, 0] PM=[0, 1, 0, 0, 0, 0, 0] PP=[0, 0, 1, 1, 1, 0] BA1=0 BS=[0, 0, 0, 0, 0] BP=[0, 0, 0, 0, 0, 0, 0, 0]

VUS: Variants uncertain significance.
